# AI‐enhanced micro‐ultrasound improves detection of clinically significant prostate cancer at biopsy

**DOI:** 10.1002/bco2.70133

**Published:** 2026-02-05

**Authors:** Muhammad Imran, Wayne G. Brisbane, Li‐Ming Su, Jason P. Joseph, Wei Shao

**Affiliations:** ^1^ School of Data Science & Analytics Kennesaw State University Marietta Georgia USA; ^2^ Department of Medicine University of Florida Gainesville Florida USA; ^3^ Department of Urology University of California Los Angeles California USA; ^4^ Department of Urology University of Florida Gainesville Florida USA

**Keywords:** artificial intelligence, diagnostic accuracy, micro‐ultrasound, point‐of‐care imaging, prostate cancer

## Abstract

**Objective:**

This study aimed to evaluate the diagnostic accuracy of artificial intelligence (AI)–enhanced micro‐ultrasound (micro‐US) for detecting clinically significant prostate cancer (csPCa) in men referred for prostate biopsy.

**Patients and Methods:**

We retrospectively analysed 145 men undergoing micro‐US‐guided biopsy (79 with csPCa, 66 without). Deep features were extracted from 2D micro‐US slices using a self‐supervised convolutional autoencoder and classified with a random forest model under fivefold cross‐validation. Patients were considered csPCa‐positive if ≥8 consecutive slices were predicted positive. Diagnostic performance was assessed against biopsy pathology using receiver operating characteristic (ROC) analysis.

**Results:**

The AI–micro‐US model achieved an area under the ROC curve (AUC) of 0.871. At a fixed threshold, sensitivity was 92.5% and specificity 68.1%, outperforming a clinical model based on prostate‐specific antigen (PSA), digital rectal examination (DRE), age, and prostate volume (AUC 0.753; sensitivity 96.2%, specificity 27.3%).

**Conclusion:**

AI‐enhanced micro‐US reduces false positives from conventional screening tools while preserving high sensitivity. It shows promise as a point‐of‐care alternative to MRI, integrating risk stratification and biopsy guidance into a single platform.

## 1 INTRODUCTION

Prostate cancer is the most common solid malignancy in men worldwide and remains a leading cause of cancer‐related mortality.^1^ Early detection of clinically significant prostate cancer (csPCa; Gleason Grade Group ≥2) is essential to preserve curative opportunities.^2^ However, current frontline screening with prostate‐specific antigen (PSA) and digital rectal examination (DRE) is limited by poor specificity, resulting in unnecessary biopsies and overdiagnosis of indolent disease.^3^, ^4^

Multiparametric magnetic resonance imaging (mpMRI) has improved csPCa detection and reshaped diagnostic pathways.^5^ However, its high cost, limited availability, and reliance on radiologic expertise constrain widespread adoption, particularly in community and resource‐limited settings. Similarly, blood and urine‐based biomarkers have emerged as adjunct tools to reduce unnecessary biopsy, but they increase cost, prolong decision‐making, and do not provide real‐time anatomical guidance for targeted sampling.

Micro‐ultrasound (micro‐US), a 29‐MHz imaging modality, enables real‐time visualization of prostate microarchitecture at a resolution superior to conventional transrectal ultrasound and comparable to mpMRI.^6^ The multicenter OPTIMUM trial recently demonstrated that micro‐US‐guided biopsy is noninferior to MRI‐targeted biopsy for csPCa detection.^7^ Despite this promise, clinical uptake of micro‐US has been limited by operator dependency and interpretive variability.^8^

Artificial intelligence (AI) offers a potential solution by standardizing interpretation, reducing subjectivity and enabling reproducible, point‐of‐care triage and biopsy guidance. In this study, we evaluated whether an AI‐enhanced micro‐US model could improve csPCa detection in men referred for biopsy after elevated PSA or abnormal DRE. Our goal was to evaluate whether AI‐enhanced micro‐US improves csPCa detection in men referred for biopsy by reducing false positives from current screening tools and to contextualize its performance relative to established biomarkers, while remaining seamlessly integrated into existing urology workflows.

## 2 PATIENTS AND METHODS

### 2.1 Patient population and data description

This retrospective study was approved by the University of Florida Institutional Review Board. We included 145 men who underwent micro‐US–guided prostate biopsy. All patients had standard clinical indications for biopsy, including elevated PSA and/or abnormal DRE. Most patients also underwent systematic 12‐core biopsy, including those without visible micro‐US lesions.

During biopsy, the operator‐recorded needle trajectories and target locations using micro‐US images, enabling retrospective mapping of biopsy cores to corresponding regions. All patients provided informed consent. Histopathological analysis of biopsy specimens served as the reference standard. Patients were classified as csPCa‐positive if any core contained Gleason score ≥3 + 4. Baseline cohort characteristics are summarized in Table 1.

**TABLE 1:** Baseline characteristics of the study cohort. Values are presented as medians (interquartile ranges, IQRs) or counts (%).

Characteristic	Positive cases	Negative cases
Age (year), median (IQR)	70 (66–74)	69 (63–71)
PSA (ng/mL), median (IQR)	8.2 (5.8–13.1)	5.7 (3.3–7.5)
DRE = 1, *n* (%)	39 (49.4%)	6 (9.1%)
DRE = 0, *n* (%)	40 (50.6%)	60 (90.9%)
Prostate volume (mL), median (IQR)	37.5 (31.5–49.4)	47.1 (39.0–55.4)
**Total**	79	66

#### 2.1.1 Micro‐US imaging

Pre‐biopsy micro‐US scans were acquired using a 29‐MHz transrectal system (ExactVu, Exact Imaging, Markham, Canada) by an experienced urologist (WGB) with 4 years of micro‐US interpretation experience. Scans were recorded at 10 frames per second for up to 30 s, producing approximately 200 to 300 2D micro‐US slices per scan.

#### 2.1.2 Clinical biomarkers

For each patient, we collected the following clinical biomarkers: PSA level, DRE findings, age, and prostate volume. Prostate volume was estimated from the pre‐biopsy micro‐US scan. We used the MicroSegNet model^9^ to segment the prostate capsule on each 2D slice and reconstructed the segmentations into a 3D volume using the method described in previous works.^10^ The final prostate gland volume (in mL) was computed from the reconstructed 3D model.

#### 2.1.3 Slice‐level labelling for model training

Model training required slice‐level annotations indicating the presence or absence of csPCa on each 2D micro‐US slice. For csPCa‐negative patients, all slices were labelled as negative. For csPCa‐positive patients, operator‐recorded needle trajectories were used to cognitively map each biopsy core to the corresponding region on the pre‐biopsy micro‐US scan. An expert urologist (WGB) manually reviewed slices surrounding the operator‐recorded trajectories to assess the extent of csPCa involvement, using sonographic features defined in the PRI‐MUS protocol.^11^ Although MRI–ultrasound fusion was not used, the urologist was aware of the MRI region of interest during scanning.^12^ Thus, lesion localization was performed based on micro‐US imaging characteristics and acoustic features consistent with cancer. Slices with suspicious sonographic features were labelled as positive. All other slices in csPCa‐positive cases were excluded from training, as their cancer status could not be confidently determined. In total, 2062 positive and 14,769 negative slices were included. Model evaluation was conducted at the patient level, using biopsy‐confirmed csPCa status to reflect clinically meaningful outcomes.

### 2.2 Model development and evaluation

#### 2.2.1 Micro‐US image feature extraction

**FIGURE 1:**
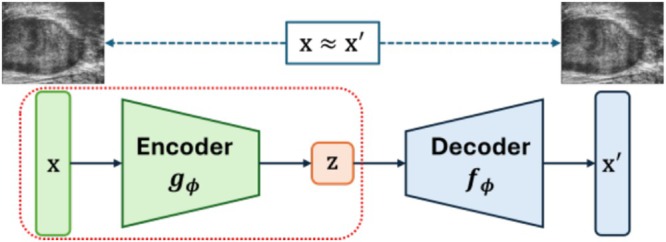
Architecture of the autoencoder used for feature extraction.

We trained a convolutional autoencoder with self‐supervised learning to extract high‐level features from micro‐US images. The autoencoder (Figure 1) consists of two symmetric components: a convolutional encoder *g*_*φ*_ and a decoder *f*_*φ*_. The encoder compresses the input image **x** into a lower dimensional latent representation **z**, while the decoder attempts to reconstruct the original image from **z**. The encoder includes five convolutional layers with increasing channel dimensions (from 3 to 256), interleaved with ReLU activations and strided convolutions for spatial downsampling. The decoder mirrors this structure with transposed convolutions and corresponding up‐sampling layers to produce the reconstructed image. The autoencoder was trained to minimize the mean squared error between the input image **x** and the reconstructed output **x**′. After training, we used the encoder as a fixed feature extractor. Each 2D micro‐US slice was passed through the encoder to generate a feature map, which was then reduced via global average pooling to obtain a 256‐dimensional feature vector.

The encoder is trained to extract meaningful features from micro‐ultrasound images by compressing them into a compact representation. During training, the decoder reconstructs the original image to ensure that the encoder captures the most informative features. After training, the decoder is discarded, and only the encoder is retained for feature extraction. This process helps optimize the encoder without requiring the decoder during model deployment.

#### 2.2.2 Slice‐ and patient‐level classification

We trained random forest classifiers to classify individual micro‐US slices as csPCa‐positive or negative using 256‐dimensional feature vectors extracted by the autoencoder's encoder, which captured key imaging characteristics such as texture, intensity and shape. Slice‐level predictions were aggregated to produce patient‐level classifications. A patient was classified as csPCa‐positive if at least eight consecutive slices were predicted positive. This rule was based on retrospective analysis of lesion length, which showed that csPCa typically spanned eight adjacent slices on average. Requiring spatially contiguous predictions helped reduce false positives and improved specificity without compromising sensitivity.

#### 2.2.3 Classification with clinical biomarkers

For comparison, we trained a random forest classifier using only clinical biomarkers: patient age, PSA level, prostate volume, and DRE outcome. Since the scikit‐learn implementation supports internal out‐of‐bag (OOB) validation, a separate validation set was used for hyperparameter tuning.

#### 2.2.4 Cross‐validation strategy

We used a fivefold internal cross‐validation strategy to ensure robust and unbiased model evaluation. The entire patient cohort (*N* = 145; 79 csPCa‐positive, 66 csPCa‐negative) was randomly divided into five equally sized, nonoverlapping folds. The model was trained and evaluated five times, each time holding out a different fold as the test set, using another fold for validation and training on the remaining three folds. This rotation ensured that every patient appeared in the test set exactly once. Model training was monitored on the validation set to prevent overfitting. The final performance metrics represent the average of results obtained from the five independent test sets, providing a stable and reliable estimate of model performance.

#### 2.2.5 Performance metrics

We evaluated model performance at the patient level using the following metrics: area under the receiver operating characteristic curve (AUROC), accuracy, sensitivity, specificity, precision, and F1‐score. All metrics were averaged across the five cross‐validation folds. AUROC served as the primary evaluation metric, as it captures overall discriminatory performance across all classification thresholds. The remaining metrics were computed using a fixed probability threshold of 0.15 for each slice. If the model predicts a probability above 0.15, the slice is considered positive. This threshold was empirically selected to balance sensitivity and specificity and is consistent with recent MRI nomogram designs,^13^ where a similar risk threshold guides biopsy decisions. While the model identifies regions with increased risk of csPCa, it does not explicitly delineate lesion boundaries. Instead, it relies on spatially contiguous positive slices to infer lesion presence, which may still require operator interpretation for localization.

#### 2.2.6 Implementation details

All autoencoder models were implemented in PyTorch (v1.13) and trained on an NVIDIA A100 GPU using the Adam optimizer (learning rate = 0.001, batch size = 32). After training, the decoder was discarded, and the encoder was used as a fixed feature extractor. Each random forest model was trained with 1000 trees, class‐balanced weights and stratified sampling to preserve the distribution of positive and negative slices. Records with missing clinical values or duplicate patient entries were removed to ensure data integrity.

## 3 RESULTS

### 3.1 Receiver operating characteristic (ROC)‐based comparison of imaging and clinical models

**FIGURE 2:**
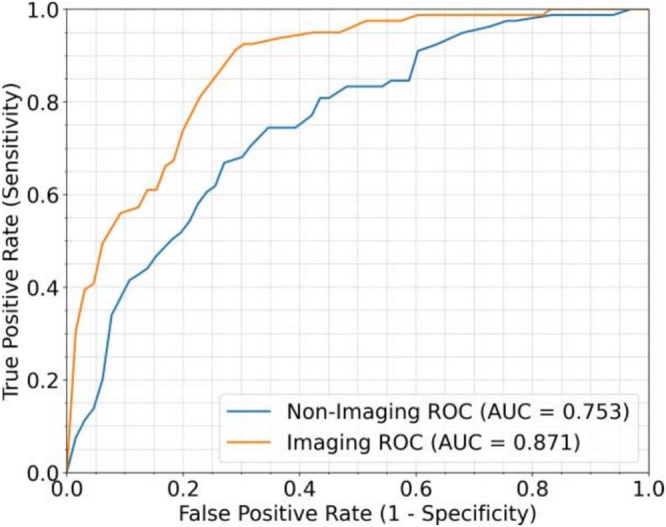
Receiver operating characteristic (ROC) curves comparing the AI‐enhanced micro‐US model (AUROC 0.871) with the clinical model (AUROC 0.753). The AI‐enhanced micro‐US model demonstrated superior discriminative performance.

Figure 2 compares the performance of the AI‐enhanced micro‐US model with the clinical model. The clinical model, trained on age, PSA, DRE, and prostate volume, achieved a mean AUROC of 0.753, indicating only moderate discriminative ability. In contrast, the AI‐enhanced micro‐US model achieved a higher AUROC of 0.871, reflecting stronger ability to distinguish csPCa from non‐csPCa based on deep imaging features extracted from micro‐US slices.

### 3.2 Comparison of classification metrics

Table 2 summarizes the average classification metrics across five cross‐validation folds. The clinical model achieved very high sensitivity (96.2%) but extremely low specificity (27.3%), resulting in a high false‐positive rate. Its precision (61.4%), F1‐score (74.9%) and accuracy (64.8%) were modest.

**TABLE 2:** Threshold‐based classification metrics (averaged across five folds) using a fixed decision threshold of 0.15.

Model	Sensitivity	Specificity	Accuracy	Precision	F1‐score
Clinical	**96.2%**	27.3%	64.8%	61.4%	74.9%
Micro‐US	92.5%	**68.1%**	**81.4%**	**77.8%**	**84.5%**

By contrast, the AI‐enhanced micro‐US model maintained high sensitivity (92.5%) while substantially improving specificity (68.1%). It also achieved higher precision (77.8%), F1‐score (84.5%) and accuracy (81.4%). This demonstrates a more balanced trade‐off between sensitivity and false‐positive control compared with the clinical model.

The AI‐enhanced micro‐US model reduced false positives compared with clinical biomarkers alone while preserving high sensitivity. These findings suggest that AI‐enhanced micro‐US can serve as a supplementary step after conventional screening tools, helping to triage patients with abnormal PSA or DRE and providing real‐time anatomical guidance for biopsy at the same visit.

## 4 DISCUSSION

This study demonstrates that AI‐enhanced micro‐US improves csPCa detection compared with traditional clinical screening variables. The clinical model, which included PSA, DRE, prostate volume, and age, achieved high sensitivity (96.2%) but very low specificity (27.3%), consistent with longstanding concerns about overdiagnosis and overtreatment associated with PSA‐based screening.^3^, ^4^ In contrast, the AI‐enhanced micro‐US model preserved high sensitivity (92.5%) while more than doubling specificity (68.1%), offering a more balanced approach that reduces false positives and unnecessary biopsies while maintaining effective csPCa detection. The inclusion of prostate volume in our clinical model likely enhances its predictive performance. Traditional models, such as the Prostate Biopsy Collaborative Group (PBCG) risk calculator, often exclude prostate volume due to its unavailability prior to MRI.^14^ Our clinical model benefits from micro‐US–based volume estimation, which is available at the time of biopsy. Although we did not include a direct comparison with PBCG, our model's AUROC is comparable to MRI‐informed nomograms,^15^ suggesting similar clinical utility.

The clinical significance of these findings lies in the potential of AI to unlock the diagnostic value of micro‐US, a low‐cost, portable, point‐of‐care modality.^6^, ^16^ As shown in the OPTIMUM trial, micro‐US alone is comparably accurate to mpMRI for guiding prostate biopsy.^7^ Yet, its clinical adoption has been limited by inter‐reader variability and interpretive complexity.^8^ Our model addresses this limitation by automating cancer detection based on high‐resolution image features, reducing subjectivity and enabling reproducible decision‐making at the point of care.

Prior efforts in AI and micro‐US, such as TRUSformer^17^ and TRUSWorthy,^18^ have focused on identifying prostate cancer from small image patches sampled around biopsy‐confirmed cancer regions. These models relied on weak labels derived from needle locations rather than true cancer boundaries. As a result, their predictions may reflect regions adjacent to cancer rather than the cancer itself, limiting clinical trust and interpretability. In contrast, our study uses slice‐level micro‐US images that have been manually aligned with histopathology‐confirmed cancer locations. This enables the model to learn from regions directly annotated as malignant, providing a more robust and clinically relevant signal. We further aggregate predictions across consecutive slices, creating a third dimension for architecture encoding and feature extraction.

The implications are significant: AI‐enhanced micro‐US can supplement current screening tools by reducing false positives from PSA and DRE while providing real‐time anatomical guidance for biopsy in the same session. Its diagnostic accuracy is comparable to that reported for urinary biomarkers, but with the added benefit of immediate point‐of‐care imaging. The portability and relatively low cost of micro‐US make it particularly valuable in community and underserved settings where MRI access is limited.

Nonetheless, these findings should be interpreted in light of several limitations. First, the study cohort consisted of patients prescreened with MRI at a single high‐volume academic centre, which may introduce selection bias. Second, the patient‐level threshold of eight consecutive positive slices was empirically derived and may require refinement in larger prospective cohorts. Third, although fivefold cross‐validation reduced the risk of overfitting, external validation in diverse populations remains essential and is underway through the prospective multicentre MUSIC‐Screen trial (NCT06626022). Finally, formal cost‐effectiveness analyses will be needed to define the real‐world value of this approach. If validated, AI‐enhanced micro‐US could provide a scalable, affordable and interpretable solution for early detection of csPCa, bridging the gap between low‐specificity biomarker screening and high‐cost MRI‐based diagnostics.

In summary, AI‐enhanced micro‐US improves specificity without compromising sensitivity and offers a scalable, operator‐independent tool to complement existing PSA‐ and biomarker‐based pathways. By bridging the gap between low‐specificity screening tests and high‐cost MRI, AI‐enhanced micro‐US has the potential to reduce unnecessary biopsies and make accurate prostate cancer detection more accessible.

## 5 CONCLUSION

This study demonstrates that AI‐enhanced micro‐US improves detection of clinically significant prostate cancer by reducing false positives from conventional screening tools while maintaining high sensitivity. By integrating risk stratification and biopsy guidance into a single, operator‐independent platform, it offers a cost‐effective, point‐of‐care supplement to existing diagnostic pathways. With prospective multicenter validation, AI‐enhanced micro‐US could make prostate cancer detection more accurate, accessible and scalable, ultimately improving patient outcomes while minimizing unnecessary biopsies.

## AUTHOR CONTRIBUTIONS

**Muhammad Imran:** methodology, software, validation, formal analysis, investigation, writing – original draft, visualisation. Wayne G. Brisbane: conceptualisation, resources, data curation, writing – review and editing, supervision. Li‐Ming Su: resources, writing – review and editing, supervision. Jason P. Joseph: resources, writing – review and editing, supervision. Wei Shao: conceptualisation, methodology, resources, data curation, writing – original draft, supervision, project administration, funding acquisition.

## CONFLICT OF INTERESTS

Wei Shao, on behalf of all co‐authors, certifies that all conflicts of interest, including specific financial interests and relationships and affiliations relevant to the subject matter or materials discussed in the manuscript (e.g., employment/affiliation, grants or funding, consultancies, honoraria, stock ownership or options, expert testimony, royalties or patents filed, received or pending), are the following: None.
